# Hesperidin Interacts With CREB-BDNF Signaling Pathway to Suppress Pentylenetetrazole-Induced Convulsions in Zebrafish

**DOI:** 10.3389/fphar.2020.607797

**Published:** 2021-01-11

**Authors:** Pallavi Sharma, Savita Kumari, Jatin Sharma, Rituraj Purohit, Damanpreet Singh

**Affiliations:** ^1^ Pharmacology and Toxicology Laboratory, CSIR-Institute of Himalayan Bioresource Technology, Palampur, India; ^2^ Academy of Scientific and Innovative Research (AcSIR), CSIR-Institute of Himalayan Bioresource Technology, Palampur, India; ^3^ Structural Bioinformatics Laboratory, Biotechnology Division, CSIR-Institute of Himalayan Bioresource Technology (CSIR-IHBT), Palampur, India

**Keywords:** brain-derived neurotrophic factor, clonic-like seizures, epilepsy, *c-fos*, flavanone glycoside, *in silico* docking, *il-10*, zebrafish larva

## Abstract

Hesperidin (3,5,7-trihydroxyflavanone 7-rhamnoglucoside) is a β-7-rutinoside of hesperetin (4′-methoxy-3′,5,7-trihydroxyflavanone), abundantly found in citrus fruits and known to interact with various cellular pathways to show a variety of pharmacological effects. The present study was envisaged to understand the anticonvulsant effect of hesperidin in a zebrafish model of pentylenetetrazole (PTZ)-induced convulsions, with the support of *in silico* docking. Healthy zebrafish larvae were preincubated with hesperidin (1, 5, and 10 µM) for 1 h, before PTZ exposure. Hesperidin treatment significantly increased the seizure latency and minimized PTZ-induced hyperactive responses. A significant reduction in *c-fos* expression further supported the suppression of neuronal excitation following hesperidin incubation in the larvae exposed to PTZ. The treatment also modulated larval *bdnf* expression and reduced the expression of *il-10*. The results of *in vivo* studies were further supported by *in silico* docking analysis, which showed the affinity of hesperidin for the N-methyl-d-aspartate receptor, the gamma-aminobutyric acid receptor, Interleukin 10 and the TrkB receptor of brain-derived neurotrophic factor. The results concluded that hesperidin suppresses PTZ-mediated seizure in zebrafish larvae through interaction with the central CREB–BDNF pathway.

## Introduction

Epilepsy is a chronic brain disorder affecting the lives of around 70 million people worldwide, and around 23 million people in Asia alone (Trinka et al., 2019). In many cases, epileptic patients are bound to suffer from seizure-related disabilities, comorbidities, and social stigma (Cho et al., 2017). Health issues like anxiety, depression, dementia, migraine, cardiac problems, peptic ulcers, and arthritis are eight times more prevalent in epileptic patients than in healthy population (Keezer et al., 2016). A vast pool of antiepileptic drugs (AEDs) is available, and a large number of patients depend on them for seizure control. However, in some cases, the conventional AEDs found to be ineffective and worsen comorbid conditions due to their low therapeutic index and alteration with neuronal processes (Henning et al., 2019; Sarangi et al., 2019).

Continuous research is in progress to explore possible alternatives for the management of epilepsy due to the fallibility and side-effects of existing AEDs. In this regard nutraceuticals are being studied as a promising intervention in addition to the available conventional drugs. Dietary flavonoids constitute one such important class of pharmacologically active compounds that have shown positive results in the management of epilepsy and associated comorbid conditions (Sharma et al., 2020). Hesperidin (3,5,7-trihydroxyflavanone 7-rhamnoglucoside) is a β-7-rutinoside of hesperetin (4′-methoxy-3′,5,7-trihydroxyflavanone), richly found in citrus fruits such as lemon, sweet orange, and grapefruits. Hesperidin possesses a variety of pharmacological activities like anticancer, anti-inflammatory, antihypercholesterolemic, antihyperlipidaemic, antihypertensive, diuretic, antiviral, calcium channel blocker activity, and many more (Hajialyani et al., 2019). Hesperidin, following ingestion, undergoes breakdown to a more readily absorbable form by the intestinal microflora. Despite reports suggesting its poor absorption and quick elimination in the human body, hesperidin shows a prolonged absorption phase and a half-life of 6 h (Li and Schluesener, 2017). Hesperidin showed no toxic, mutagenic, or carcinogenic effects when administered up to 5% in the diet, following 13 weeks of its exposure in mice. Hesperidin was found to be safe and devoid of side effects even during pregnancy (Garg et al., 2001). Besides this, its exposure turned out to be completely safe in zebrafish larvae, and no toxic effects were observed after 96 h of exposure (Ali et al., 2012).

Hesperidin and its aglycone Hesperitin, are known to surpass blood brain barrier and show neuroprotective effect in both *in vitro* and *in vivo* studies (Roohnakhsh et al., 2014). Hesperidin treatment was found to increase the neuronal population *in vitro*. Also a culture of neural progenitor cells conditioned with hesperidin treated astrocytes showed a higher number of neural progenitors and post-mitotic neurons (Nones and Gomes., 2011; Nones et al., 2012). In another study, hesperidin decreased the excitatory responses of 4-aminopyridine and bicuculline in a rat hippocampal preparation. Whereas hesperetin was found to be effective against tetraethylammonium and pentylenetetrazole (PTZ) induced hyperexcitatory responses in the same preparation (Dimpfel, 2006). Kwon et al. (2018), reported that oral administration of hesperetin delays the onset of seizures triggered by kainic acid in a mouse model of temporal lobe epilepsy with attenuation of pro-inflammatory cytokine expression and granule cell dispersion in the hippocampus. Kumar et al. (2013) demonstrated that hesperidin (100 mg/kg) administration in PTZ treated mice augmented the antiseizure and neuroprotective effect of Diazepam (0.2 mg/kg) and Gabapentin (10 mg/kg). In a study performed on zebrafish, 28 mg/mL of aqueous extract of *C. aurantium* leaves containing hesperidin, neohesperidin dihydrochalcone, and neohesperidin increased the seizure latency by a mechanism involving N-methyl-d-aspartate (NMDA) and mGluR's (metabotropic glutamate receptors) I and II (Rosa-Falero et al., 2015). In another study intraperitoneal administration of hesperidin in rats attenuated the kainic acid-induced extracellular glutamate release and neuronal loss in the hippocampal CA3 area, suggesting that hesperidin can cross the blood-brain barrier *per se* (Chang et al., 2015). Huang et al. (2012) studied the neuroprotective effect of hesperetin and hesperidin against A-β1-42 induced autophagy and impairment of glucose utilization in Neuro-2A cells. They concluded that both hesperidin and hesperitin were substantially effective.

Despite the studies conducted using different seizure models by various research groups, the precise mechanisms of seizure suppression by hesperidin remain unclear. The current study aimed to get a more in-depth insight into the antiseizure effect of hesperidin. *In silico* analysis was performed to understand the intramolecular interactions of hesperidin with primary identified targets *viz:* NMDA (N-methyl-d-aspartate), TrK, GABA (gamma-aminobutyric acid) and IL10 (Interleukin10). Zebrafish (*Danio rerio*) model of PTZ-induced seizures and hyperactive response analysis was used to study the effect of hesperidin. The findings of *in silico* and *in vivo* studies were further corroborated with the gene expression analysis to understand the antiseizure mechanism of hesperidin.

## Experimental Procedures

### Drugs and Chemicals

Hesperidin was obtained from TCI Chemicals, Japan. PTZ, SYBR green jumpstart Taq Ready mix kit, and Trizol reagent procured from Sigma Aldrich, United States. Applied Biosystems, United States provided High capacity cDNA-RT kit. Whereas, RNase-free DNase kit was procured from Promega, Madison, United States. Sea salt was obtained from Aquarium Systems, Germany.

### Protein Ligand Interactions

Structural 3D co-ordinates of the receptor proteins, namely NMDAR (pdb id: 5EWJ), TRK (pdb id: 5JFW), GABA (pdb id: 6D6T), and Interleukin-10 (pdb id: 2ILK) were acquired from the Protein Data Bank (Berman, 2000). Similarly, the spatial data file for hesperidin was obtained from PubChem (Kim et al., 2019). Gaussian 16 minimization (with DFT) protocol used to optimize the geometry of the selected molecule (Zheng and Frisch, 2017). The Discovery studio package utilized to define protein preparation, missing loop region-building, and ionization states (Biovia et al., 2000). The molecular docking experiment performed using FlexX (version 4.1) on the receptor proteins utilizes an incremental construction algorithm for physicochemical design (Rarey et al., 1996a; Rarey et al., 1996b). This algorithm is differentiated into 3 parts viz: scoring function, conformational space model, and several interaction models of the protein-ligand complex. MIMUMBA database was applied to allocate respective non-cyclic bonds with an array of minimum energy torsion angles, followed by an analysis of intra-molecular conflicts and application of the FlexX scoring function (Rarey et al., 1997). These scoring functions minutely altered for LUDI configuration via Böhm. The preparation protocol of the pdb structures on LeadIT was as follows: chain A of TRK (5JFW), chain B of NMDAR (5EWJ), chain D of GABA (6D6T), and chain A of Interleukin-10, selected for the docking experiments. The reference ligands in the binding pockets designated for the 4 selected protein receptors, where 6K2 chosen in the case of TRK, QEL for NMDAR, and FYP for GABA.

The reference ligands serve to provide the active binding site that contains all the required amino acids where it should bind. In the case of Interleukin-10 a reference ligand was not present, so we identified a binding pocket in its conformation via the binding pocket identification tool of LeadIt. This identified binding pocket also contained the essential amino acids required for the binding of a ligand. The remaining settings during the receptor preparation step kept default. The ligand structure for docking stored in “.mol2” format file and uploaded onto leadIT during the docking experiments. Ten poses were generated for the ligand during docking, keeping all the parameters like the scoring function and the docking strategy as default. Pose generation was followed by estimating the binding energy and the ligand efficiency through the hydrogen dehydration (HYDE) scoring function (Schneider et al., 2013). The octanol-water partition coefficient was applied to align conditions for small molecules (Reulecke et al., 2008). HYDE calculations, 2D, and 3D interaction diagrams for the protein-ligand complex were obtained using the LeadIT software (Stierand and Rarey, 2007; Stierand and Rarey, 2010).

### Molecular Dynamics Simulation and MMPBSA

After that, a 50 ns long molecular dynamic (MD) simulation analysis on the 4 protein-ligand complexes was performed via the GROMOS96 43al force field (Chiu et al., 2009), with the help of the PRODRG server and GROMACS 5.0.7 for topology development process (Schüttelkopf and Van Aalten, 2004). An electrically steadied solvation box organized for the complexes. The stability attained by addition of 2 CL-1 ions by the “gmx genion” script. Further, energy minimization methodologies of GROMACS, and the MD simulations conducted in 2 steps, a canonical ensemble and a constant temperature and pressure ensemble, which was achieved by the Berendsen thermostat (Berendsen et al., 1984). The Shake algorithm runs for a time step of 2 fs that limits the H-bond lengths and trims the long-range interactions at 1.0 nm. The residues also demarcated at a certain pH at which histidine considered to be neutral, and the results examined using scripts like “gmx rms”, “gmx rmsf”, and “gmx h bond” of GROMACS that provided the RMSD, RMSF, and the H-Bonds of the formed complexes (Van Der Spoel et al., 2005). Finally, the stable conformations of the protein-ligand complexes based on the simulation results subjected to Molecular mechanics Poisson-Boltzmann surface area (MMPBSA) calculations (Kollman et al., 2000) via the script “g_mmpbs” (Kumari et al., 2014) of GROMACS v5.0.7.

### Animals and Ethics

Zebrafish (wild-type) were maintained in the standard aquatic environment inside a Stand-Alone System (Tecniplast, Buguggiate, Varese, Italy). The circulating system water provided continuous aeration and four-stage filtration. The system water pH, conductivity, and temperature were set at 7–7.5, 400–600 μS, and 26–28°C, respectively. Fish were fed twice daily with freshly hatched live Artemia (Inve Aquaculture, Inc., Salt LakeCity, United States). The light: dark cycle of the fish room was set at 14:10 h. Individual breeding tank was maintained for breeding in the ratio of 4 females: 2 males to collect healthy embryos, as described earlier (Tanwar et al., 2019). The collected embryos were cleaned and incubated in a biological oxygen demand incubator (Relitech, Ambala, India) in Petri dishes until 7 *dpf* (*days post-fertilization*) with regular water change during the incubation period. The study protocol was approved by the Institutional Animal Ethics Committee (IAEC) of CSIR–IHBT established under Committee for the Purpose of Control and Supervision of Experiments on Animals, Ministry of Fisheries Animal Husbandry and Dairying, Department of Animal Husbandry and Dairying, Government of India.

### PTZ-Induced Seizures in Larva

The study was performed in a transparent 6 well flat-bottomed plate with an individual well size of 3.5 cm (dia) × 2 cm (depth). The larvae at 7 dpf were divided into 3 different groups (*n* = 8) and incubated with 1, 5 and 10 µM of hesperidin (selected based on our pilot studies conducted at different concentrations), represented as *hes1/ptz, hes5/ptz*, and *hes10/ptz,* respectively. Two separate groups of larvae served as naïve, and vehicle control groups indicated as *veh/naive* and *veh/ptz,* respectively, and incubated in system water. After an hour of incubation, the larvae of *hes1/ptz, hes5/ptz, hes10/ptz*, and *veh/ptz* groups were placed individually in wells filled with PTZ (8 mM). Latency to the Stage 3 seizures was noted, and the upper cut off time was fixed at 15 min. The solution was maintained at 28 ± 1°C and changed after each recording. A three-point scale was applied for scoring purpose as, Stage 1: increased abnormal swimming activity; Stage 2: circular whirlpool-like activity and; Stage 3: clonus-like seizures accompanied by single sided or complete loss of posture (Baraban et al., 2005). Hyperactive responses recorded for the first 5 min as a measure of total distance traveled and mean velocity using a camera (c922 Pro Stream, Logitech, Hong Kong) connected to a video tracking system (SMART V.3.0. Panlab Barcelona), as described earlier (Kumari et al., 2019). The recordings of the *veh/naive* group was made in system water. Furthermore, the latency to the onset of Stage 3 seizures was also noted in all the groups exposed to PTZ. All the recordings were made by an observer blind to the treatments.

### Gene Expression Studies

Gene expression studies were carried out in the treated larvae exposed to PTZ. The larvae at 7 *dpf* incubated in system water and 10 µM hesperidin (highest effective concentration) in different groups *veh/ptz-s* and *hes10/ptz-s*, respectively. Each group consisted of 3 sets of larvae (*n* = 20/set). After 1 h of the incubation, all the larvae were exposed to PTZ (8 mM) for 15 min. Similarly, a group of larvae containing 3 separate sets was kept in system water but was not exposed to PTZ represented as *veh/naïve-s*. After that, total RNA isolation from larvae was carried out through the Trizol reagent (Sigma Aldrich, United States) method, as described earlier (Tanwar et al., 2019). Chloroform was added to the homogenate and centrifuged for 15 min at 4°C (12,000 g) followed by incubation for 5 min. Thereafter, the aqueous layer was separated and treated with isopropanol to precipitate RNA and again centrifuged for 10 min at 4°C. The RNA pellet was secured carefully, given frequent washings of ethanol (75%), succeeded by centrifugation at 4°C for 5 min. Nuclease-free water was utilized to dissolve the obtained RNA pellet, which was then quantified on a Nanodrop (ND-1000 Thermo Scientific, United States). Trace amounts of DNA were removed from the obtained RNA by treating it with an RNase-free DNase kit (Promega, Madison, United States). RNA further processed for cDNA synthesis with the help of a high capacity cDNA-RT kit (Applied Biosystems, United States). Afterward, quantitative real-time polymerase chain reaction analysis was accomplished using SYBR Green Jump start Taq Ready Mix (Sigma Aldrich, United States) and *elf1α *(*elongation factor-1-α;* housekeeping gene used for normalization) of zebrafish as reference standard on Step One Plus Real Time PCR system (Applied Biosystems, United States). The annealing temperature was standardized at 55 °C for *bdnf* (Brain derived neurotrophic factor), *il-10* and *c-fos*. PCR conditions were maintained as stage I: Preheating (5 min at 94°C), stage II (40 cycles): cDNA template Denaturation (30 s at 94°C), Annealing (30 s at 55°C), Extension (30 s at 72°C), Stage III: Extension (1 min at 72°C). The experiment was performed in triplicate and repeated twice. Primers designed using Primer Express Software 3.0 (ABI, United States). The sequences for *c-fos*, *bdnf*, *il-10* and *elf1α* included [F: AAC​TGT​CAC​GGC​GAT​CTC​TT; R: GCA​GGC​ATG​TAT​GGT​TCA​GA] [F: ATA​GTA​ACG​AAC​AGG​ATG​G; R: GCT​CAG​TCA​TGG​GAG​TCC] [F: AcAG; R: GGT​CTC​CAA​GTA​GAA​ATG​CAG​G] and [F: GAT​GCA​CCA​CGA​GTC​TCT​GA; R: TGA​TGA​CCT​GAG​CGT​TGA​AG], respectively. The expression of genes analyzed by 2^ddCT method and expressed as fold change with respect to naïve (Mazumder et al., 2017).

### Statistical Analysis

The results of latency to stage 3 seizures, distance travelled and average speed expressed as mean ± Standard Error of Mean (SEM). In contrast, the results of gene expression analysis presented as a mean ± Standard Deviation. The statistical significance was determined using one-way analysis of variance followed by Tukey’s post hoc test. The results were considered significant at *p* < 0.05. SigmaStat^®^ statistical software version 4.0 was used for statistical analysis.

## Results

### Protein Ligand Interaction Analysis

**FIGURE 1:**
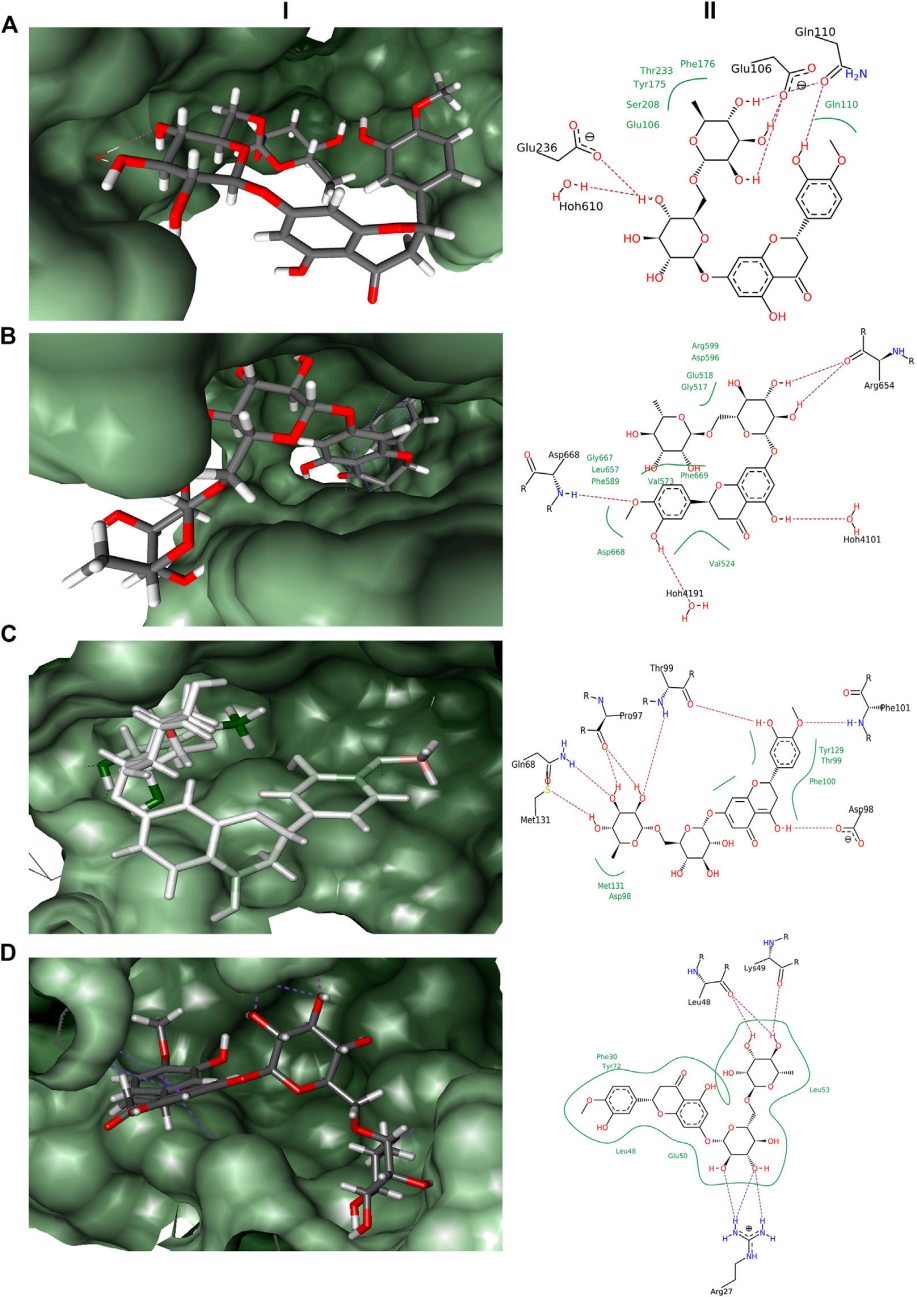
Docking results obtained from LeadIT for the four receptors with hesperidin. **(A)** 3D and 2D interaction diagram of Hesperidin with NMDAR receptor. **(B)** 3D and 2D interaction diagram of Hesperidin with TRK receptor. **(C)** 3D and 2D interaction diagram of Hesperidin with GABA receptor. **(D)** 3D and 2D interaction diagram of Hesperidin with Interleukin-10 receptor; (i) 3D pose, and (ii) 2D pose.

In the docking experiments, hesperidin was found to interact with the receptor proteins TRK, NMDAR, GABA, and interleukin-10 (Table 1). Hesperidin adopted a stable conformation in the binding pocket for all the 4 receptor proteins. The binding patterns of hesperidin with the proteins illustrated in Figure 1. Hesperidin molecule formed hydrogen bonds with the residues Glu106, Gln110, and Glu236, whereas hydrophobic interactions with residues Glu106, Gln110, Tyr175, Phe176, Ser208, and Thr233 obtained with NMDAR receptor. It additionally formed a hydrogen bond interaction with 1 water molecule, thereby resided on the final free energy of −17 kJ/mol and ligand efficiency of 0.10. The molecule hesperidin forms hydrogen bonds with residues Arg654 and Asp668, whereas hydrophobic interactions with residues Gly517, Glu518, Val524, Val573, Phe589, Asp596, Arg599, Leu657, Gly667, Asp668, and Phe669 of TRK receptor. It also formed a hydrogen bond with 2 water molecules and obtained final free energy of −40 kJ/mol and ligand efficiency of 0.22. Hesperidin also formed hydrogen bonds with the residues Gln68, Pro97, Asp98, Thr99, Phe101, and Met131, whereas hydrophobic interactions with residues Asp98, Thr99, Phe100, Tyr129, and Met131 of GABA receptor. HYDE calculations revealed the final free energy of −30 kJ/mol and ligand efficiency of 0.17 with the GABA receptor. Interestingly, no interaction found at the benzodiazepine binding site of the GABA receptors. The molecule here formed hydrogen bonds with the residues Arg27, Leu48, and Lys49, whereas hydrophobic interactions with residues Phe30, Leu48, Glu50, Leu53, and Tyr72 of Interleukin-10 receptor. From HYDE calculations, we obtained the final free energy of −23 kJ/mol and ligand efficiency of 0.13. Based on the HYDE scoring function's scores, we found that the hesperidin molecule showed varied levels of interaction with the studied receptors.

**TABLE 1:** Final free energy and ligand efficiencies of ligand molecule obtained after docking from FlexX.

Ligand	NMDAR		TRK		GABA		Interleukin-10	
	ΔG (kJ/mol)	LE	ΔG (kJ/mol)	LE	ΔG (kJ/mol)	LE	ΔG (kJ/mol)	LE
Hesperidin	−	0.10	−40	0.22	−30	0.17	−23	0.13

### Molecular Dynamic Simulation and MMPBSA Analysis

**FIGURE 2:**
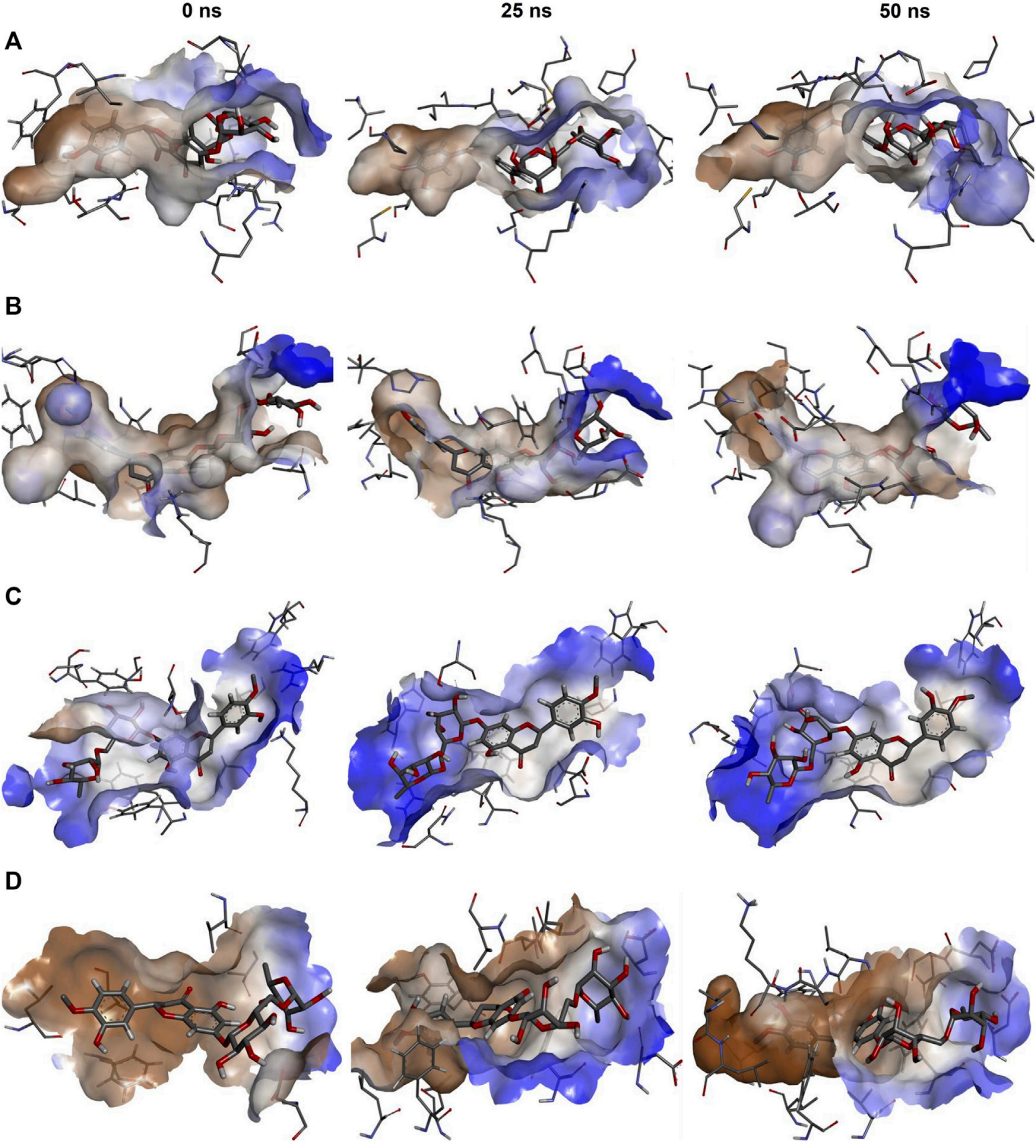
Snapshots of the complexes at 0 ns (start), 25 ns (midway), and 50 ns (end) of the simulation visualised in Discovery studio. **(A)** NMDAR-Hesperidin, **(B)** TRK-Hesperidin, **(C)** GABA-Hesperidin, and **(D)** Interleukin10-Hesperidin.

Docking analysis only provides static interaction images. Hence the dynamic nature of protein-ligand interactions cannot be fully explained by this analysis. Hence, to dynamically analyze the protein-ligand complexes, we also performed MD simulations coupled with MMPBSA calculations. The snapshots of the conformation obtained by the protein-ligand complex at 3 instances (0, 25, and 50 ns) of simulation, namely, start, midway, and end have shown in Figure 2. The figure also shows the hydrophobic interaction surface of the receptor and other interactions formed between hesperidin and the receptors. It also depicts that the ligand remained inside the receptor's binding pocket for the entire simulation period.

**FIGURE 3:**
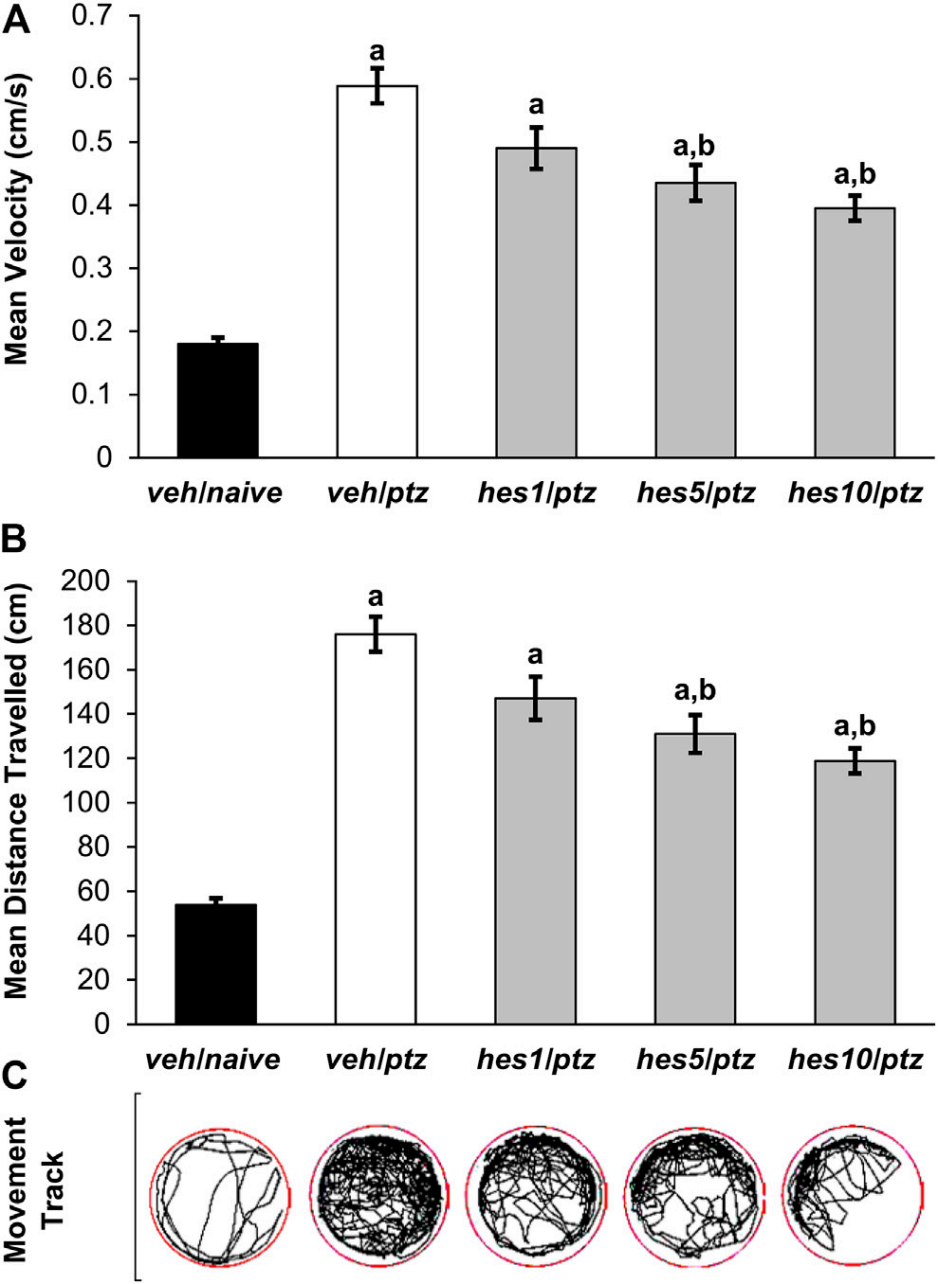
Effect of hesperidin on PTZ-induced hyperactivity in 7 *dpf* zebrafish larvae. **(A)** Mean swimming speed, **(B)** total distance travelled, and **(C)** larva movement track. ^a^ *P* < 0.05 as compared to *veh/naive* (naïve) group and; ^b^ *P* < 0.05 as compared to *veh/ptz* (vehicle control) group. The results expressed as mean ± SEM. ***veh/naive***: system water incubated group not exposed to PTZ; ***veh/ptz***: system water incubated group exposed to PTZ; ***hes1/ptz***: Hesperidin 1 μM incubated group exposed to PTZ; ***hes5/ptz***: Hesperidin 5 μM incubated group exposed to PTZ and; ***hes10/ptz***: Hesperidin 10 μM incubated group exposed to PTZ.

The simulations results provided an insight into the conformational changes taking place in the complexes with time, and these produced in the form of graphical representation for RMSD, RMSF, and H-bonds. RMSD graph depicted that the protein complexes obtained stable trajectories beyond a specific period, with low levels of fluctuations (Supplementary Material; Figure 1). Stable RMSD trajectories imply that the ligand has obtained a stable binding conformation inside the protein's binding pocket. On the contrary, the graph for RMSF of the protein showed higher levels of fluctuations for the residues of the protein (Supplementary Material; Figure 2). Higher RMSF for the residues denotes a higher level of flexibility, which implies increased potential to interact with the ligand. Lastly, the H-bonds graph depicted the number of H-bonds formed between the protein and the ligand with time (Supplementary Material; Figure 3). H-Bond formation generally linked with the stability of a ligand that achieves in the binding pocket, therefore more the number of H-bonds formed, more significant is the stability obtained. Hence, it was assumed that hesperidin forms an ample amount of H-bonds with all the proteins to maintain a stable conformation.

**FIGURE 4:**
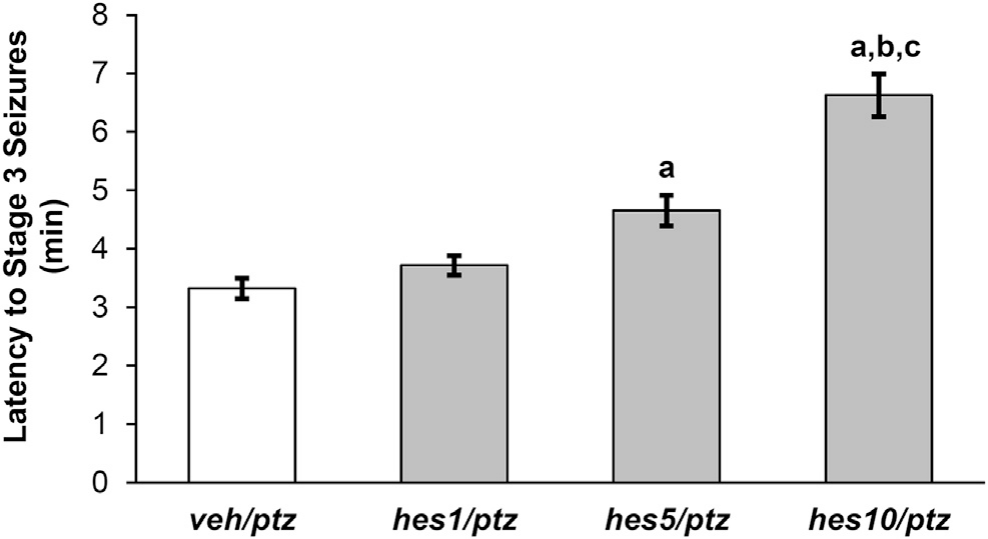
Effect of hesperidin on the latency to PTZ-induced seizures (Stage 3). ***veh/naïve*** group was not exposed to PTZ, hence not shown in the figure. ^a^ *P* < 0.05 as compared to *veh/ptz* (vehicle control) group; ^b^ *P* < 0.05 as compared to *hes1/ptz* group and; ^c^ *P* < 0.05 as compared to *hes5/ptz* group. The results expressed as mean ± SEM. ***veh/ptz***: system water incubated group exposed to PTZ; ***hes1/ptz***: Hesperidin 1 μM incubated group exposed to PTZ; ***hes5/ptz***: Hesperidin 5 μM incubated group exposed to PTZ and; ***hes10/ptz***: Hesperidin 10 μM incubated group exposed to PTZ.

Further, these complexes put through MMPBSA calculations, which provided information about the binding energy obtained by the ligand molecule with the protein and the contribution energy attained by the residues of the protein. The graphical representation of the binding energies obtained by hesperidin with the 4 protein receptors shown in Supplementary Material; Figure 4, where lower levels of binding energies depicted stronger interactions. The average binding energies and their constituent energies depicted in Table 2. It showed that hesperidin achieved lower values with NMDAR and Interleukin-10, as compared to TRK and GABA.

**TABLE 2:** Binding energy and its constituents calculated through MM-PBSA calculations for the protein-ligand complexes.

Complexes	Binding Energy (kJ/mol)	Van der Waal's Energy (kJ/mol)	Electrostatic Energy (kJ/mol)	Polar Solvation Energy (kJ/mol)	SASA Energy (kJ/mol)
NMDAR-hesperidin	−254.160	−365.438	−127.379	268.324	−29.667
TRK-hesperidin	−162.453	−339.164	−106.594	311.526	−28.221
GABA-hesperidin	−156.108	−274.592	−98.771	241.504	−24.249
Interleukin10-hesperidin	−249.671	−316.658	−98.299	191.066	−25.780

**FIGURE 5:**
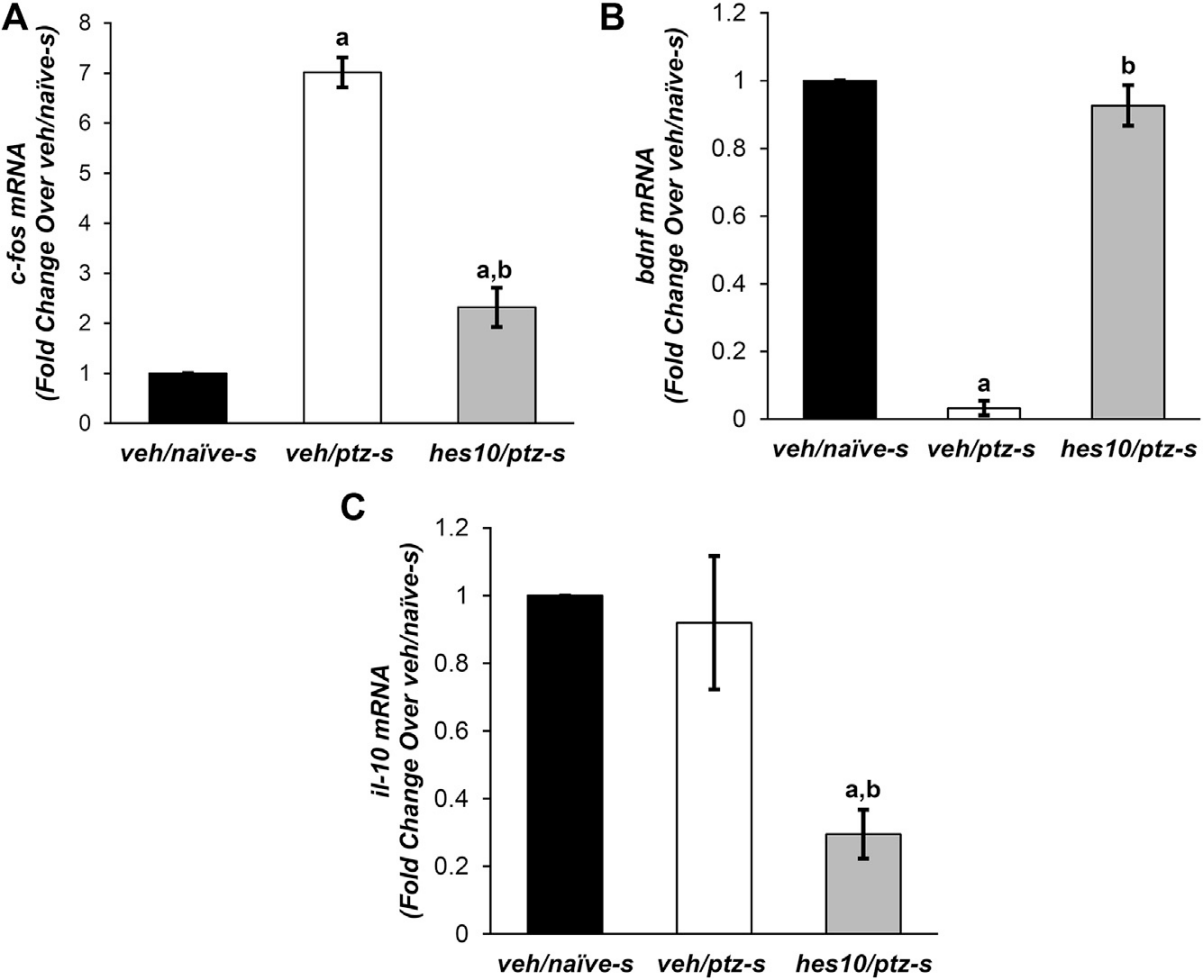
Effect of hesperidin on PTZ-mediated alterations in mRNA levels of **(A)** *c-fos*, **(B)** *bdnf*, and **(C)** *il-10*. ^a^ *P* < 0.05 as compared to *veh/naive-s* (naive) group and; ^b^ *P* < 0.05 as compared to *veh/ptz-s* (vehicle control). The results expressed as mean ± SD. ***veh/naïve-s***: system water incubated group not exposed to PTZ; ***veh/ptz-s***: system water incubated group exposed to PTZ; and; ***hes10/ptz-s***: Hesperidin 10 μM incubated group exposed to PTZ.

The contribution energies of the residues of the protein receptor toward the hesperidin were also analyzed (Supplementary Material; Figure 5). Similarly, the residues of NMDAR and Interleukin-10 showed significant levels of contribution toward interaction. While residues of TRK and GABA showed moderate levels of contribution toward hesperidin. Based on these energies, it can be correlated that hesperidin interacts strongly with NMDAR and Interleukin-10 receptors.

### Effect of Hesperidin on PTZ-Induced Hyperactive Responses in Larva

Hyperactive responses were observed in the larvae exposed to PTZ in comparison to the naïve group. A marked increase (*p* < 0.001) in the average swimming speed was observed in veh/ptz group exposed to PTZ in contrast to the larvae kept in system water alone (*veh/naïve* group). Whereas, pre-treatment with hesperidin in *hes5/ptz* (*p* = 0.001) and *hes10/ptz* (*p* < 0.001) groups showed a significant reduction in the average speed in comparison to vehicle control *veh/ptz* group (Figure 3A). Likewise, the larvae exposed to PTZ showed a significant (*p* < 0.001) upsurge in the total distance travelled in *veh/ptz* group, as compared to the non-treated larvae of *veh/naïve* group (Figure 3B). The groups pre-incubated with hesperidin at 5 µM (*p* = 0.001) and 10 µM (*p* < 0.001) showed a significant decrease in total distance travelled in comparison to *veh/ptz* group. The hyperactive responses in hesperidin treated *hes5/ptz*, and *hes10/ptz* groups were not normalized, as the total distance travelled and mean speed remained significantly (*p* < 0.001) higher in comparison to the *veh/naïve* group. However, hesperidin at 1 µM concentration showed no effect in mean speed (*p* = 0.062), and total distance travelled (*p* = 0.063) in comparison to the *veh/ptz* group.

### Effect of Hesperidin on PTZ-Induced Seizures in Larva

The larvae pre-treated with different doses of hesperidin showed a delayed appearance of stage 3 seizures as that of vehicle control group (Figure 4). The group incubated with 10 µM concentration of hesperidin showed a significant (*p* < 0.001) increase in latency to clonic-like seizures compared to the vehicle control *veh/ptz* group. Furthermore, the *hes5/ptz* group also significantly (*p* = 0.005) delayed the onset of clonic-like seizures compared to the vehicle control group. However, hesperidin at 1 µM concentration showed insignificant (*p* = 0.7) change in latency to first clonic-like seizure in comparison to veh/ptz group.

### Effect of Hesperidin on Gene Expression

*c-fos* expression was significantly (*p* < 0.001) elevated after PTZ exposure in the vehicle control *veh/ptz-s* group compared to the naïve *veh/naïve-s* group. Pre-treatment with hesperidin displayed a significant (*p* < 0.001) reduction in *c-fos* expression in comparison to *(veh/ptz-s)*, however its level remained elevated (*p* = 0.003) in *hes10/ptz-s* group in contrast to *veh/naïve-s* group (Figure 5A).

The vehicle control *veh/ptz-s* group of larva exposed to PTZ showed a marked (*p* < 0.001) decrease in bdnf expression as compared to veh/naïve-s group (Figure 5B). Interestingly, *bdnf* expression was elevated significantly (*p* < 0.001) in the group pre-incubated with 10 µM concentration Hesperidin before PTZ exposure compared to veh/ptz-s group.

There was insignificant (*p* = 0.711) change in the mRNA level of *il-10* in the vehicle control *veh/ptz-s* group compared to the naïve veh/naïve-s group. Interestingly, marked decrease in *il-10* expression was observed in *hes10/ptz-s* group pre-incubated with hesperidin in contrast to *veh/ptz-s* (*p* = 0.002) group and *veh/naïve-s* (*p* = 0.001) group (Figure 5C).

## Discussion

In the current study, it was observed that hesperidin treatment delayed the onset of stage 3 seizures in PTZ treated zebrafish larvae. Besides, a substantial reduction in the PTZ-induced hyperactivity (as inferred by total distance travelled and average swimming speed) also observed. *In silico* docking, analysis demonstrated an interaction of hesperidin with NMDA, GABA, Trk, and IL-10 receptors. There was a considerable amount of H-bonding and stable confirmation of hesperidin with all the 4 proteins in MD simulation studies. Furthermore, elevated levels of *bdnf* with reduced *c-fos* and *il-10* expression was observed in the larvae pre-incubated with hesperidin.

Zebrafish larvae at 7 *dpf* possess a fully functioning nervous system and a characteristic locomotor behavior (Mussulini et al., 2013). Baraban et al. (2005), observed that the optic tectum of PTZ treated zebrafish larvae displayed epileptiform electrographic discharges in the field potential recordings. These behaviors can be correlated with the seizure activity in mammals (Berghmans et al., 2007). Similarly, in our study, PTZ exposure led to an increase in the locomotion, followed by clonic-seizure like activity. The groups preincubated with hesperidin exhibited an increased latency to stage 3 seizures, which signifies this citrus flavonoid’s anticonvulsive action. These findings are in obedience to the previously conducted studies on hesperidin validating its antiseizure effect (Dimpfel, 2006; Kumar et al., 2013; Citraro et al., 2016). Earlier studies also revealed increased locomotor activity in larvae exposed to PTZ (Kumari et al., 2019). In the present study, pre-treatment with hesperidin resulted in a marked reduction in the hyperactive events, as shown by the diminution in the distance travelled and average speed. In previous studies, antiepileptic agents have also shown the suppression of PTZ-mediated hyperactive responses (Mazumder et al., 2019). Hence, the observed reduction in the locomotor activity in hesperidin treated larvae against PTZ can be correlated with its antiepileptic effect.

Flavonoids interact with different cell signaling pathways and receptors to show a variety of pharmacological effects. The gut bacteria deglycosylate hesperidin into hesperitin. Both hesperidin and hesperitin are capable of blocking the effects of increased calcium, and thus can be possibly effective in conditions dealing with the brain hyper-excitability (Dimpfel, 2006). The antiseizure effect of hesperidin can be correlated with enhanced GABAergic transmission in the brain (Hinton et al., 2017). Flavonoids have already been identified as a group of bioactive metabolites beneficial in CNS conditions associated with GABAergic transmission, particularly epilepsy, anxiety, depression, cognition deficit, sleep disorders, and many more (Johnston, 2015). Many conventional antiepileptic drugs also act by targeting GABAergic transmission, either by directly acting on receptors or by impeding its inactivation mechanisms (Madsen et al., 2010). PTZ itself acts as a non-competitive antagonist at the GABA receptor complex and inhibits the influx of chloride ions, causing hyperexcitability to induce seizures (Hansen et al., 2004). Hence, seizure suppression observed by hesperidin treatment can be correlated with its possible interaction with GABA receptors.

Further, our computational analysis results supported a direct action of hesperidin molecule on the GABA receptors, strengthening the observation of GABAergic action for the anticonvulsant effect. Hesperidin showed interaction with GABA receptors at the active sites other than classical benzodiazepine binding targets. The finding is in line with some previous reports, which suggest the action of flavonoids on GABA receptor through various other modulatory sites, in addition to the flumazenil sensitive benzodiazepine binding site (Fernández et al., 2005; Hanrahan et al., 2011).

Furthermore, seizures lead to enduring alterations in gene transcription in the neurons. The results revealed that PTZ exposure increased the expression of *c-fos*. *c-fos* considered as a useful marker of neuronal excitation, inflammation, injury, or trauma. It is reported that zebrafish larvae following PTZ exposure show ictal and interictal electrical activity patterns with an increase in *c-fos* expression in different regions of the brain (Hortopan et al., 2010). Studies conducted on the PTZ model have shown that antiseizure compounds reduce the expression of *c-fos* (Mazumder et al., 2019). Labinar et al. (1993) suggested that *c-fos* expression induced by seizures can be correlated with sustained neuronal depolarization, an increase in intracellular calcium levels, and increased expression of receptors, such as NMDA. Reduction in larval *c-fos* expression following hesperidin treatment in our study further supported its inhibitory action on excessive neuronal discharge.

Studies conducted in the past also stressed on the modulation of neurotrophic factors by flavonoids (Xu et al., 2013; Diniz et al., 2014). *bdnf* is a dependent gene of cAMP response element binding protein (CREB), which plays a vital role in neuroprotection and other neuronal physiological functions. Several external stimuli that activate the intracellular pathways (like activation of PI3K/Akt, Ca 2+/CAM or PLC/IP3 signaling pathway, NMDA activation) result in the phosphorylation of CREB which provokes transcription of *bdnf*. Bdnf acts through its TrkB receptor to initiate a downstream row of events (Sharma et al., 2019). Previous reports have recognized that *bdnf* is strongly expressed in the brain of 7 *dpf* zebrafish larvae (Cacialli et al., 2016). The release of Bdnf and TrkB activation is known to exert diverse effects relative to seizure. Falcicchia et al. (2018), injected Bdnf releasing cells in the hippocampus of pilocarpine treated rats and witnessed a considerable reduction in the frequency of spontaneous seizure. Antunes et al. (2016) found that hesperidin increases the brain Bdnf level in rats subjected to olfactory bulbectomy. PTZ exposure or seizure activity may influence different complex mechanisms in the cells that regulate *bdnf* expression. However, our results showed the downregulation of *bdnf* expression in larval tissue of the PTZ treated group. This can be correlated with the previous finding suggesting that *bdnf* expression can show time-dependent changes in the brain following PTZ exposure. Zhu et al. (2012), studied the effect of PTZ treatment on Bdnf expression at protein and gene level in the hippocampal astrocytes at multiple time points. It was observed that *bdnf* expression was significantly reduced after PTZ exposure and started restoring after 8 h. In our study, the group treated with hesperidin retained *bdnf* expression to a normal level, thus further supported its antiseizure effect. Previous studies showed that, following binding with TrkB, Bdnf triggers its downstream signaling cascades, ultimately resulting in modulation of neurotransmission and enhanced synaptic functions (Sandhya et al., 2013; Björkholm and Monteggia, 2016). Li et al. (2016) reported that hesperidin induces the upregulation of *bdnf* in an ERK-dependent manner. In line with the previous findings, an affinity of hesperidin was observed toward Trk receptors in our docking studies. This indicates that hesperidin is capable of potentiating the effects of Bdnf by directly acting on its receptors. Hence, it can be correlated that *bdnf* expression by hesperidin on PTZ exposure and its direct affinity toward Trk receptors might contribute to its observed anticonvulsant effect.

Alterations in the neurotransmitter functions are closely associated with epilepsy. Excessive stimulation of NMDA receptors, and deficiency of GABA, is known to trigger seizures. The computational analysis results revealed that hesperidin interacts with NMDA receptors. NMDA receptors are overstimulated by excessive glutamate release, which leads to increased intracellular Ca2+ resulting in excitotoxicity (Amiri et al., 2016). Studies have shown that NMDA antagonists can selectively suppress seizures-induced morphological changes and restrict *c-fos* expression (Labiner et al., 1993; Toth et al., 2018). Since hesperidin showed an affinity for NMDA receptors in our computational analysis, it can be correlated that the observed anticonvulsant effect is due to its interaction with this receptor. Citraro et al. (2016) showed the anticonvulsant effect of hesperidin-rich orange juice through interaction with the NMDA receptors' glycine site. NMDA receptors play a crucial role in the activation of the CREB-BDNF pathway. The modulation of NMDA by hesperidin indicates its multidimensional approach of action, which needs to be investigated thoroughly.

*In silico* docking, studies also showed the interaction of hesperidin with *il-10*. Moreover, decreased *il-10* expression was also observed in hesperidin treated zebrafish larvae. This is in line with a previous report that found human mesenchymal stem cells treated with 5 μM hesperidin reduced the expression of *il-10*, along with other proinflammatory cytokines (Xiao et al., 2018). In another study, hesperidin decreased *il-10* expression and reversed memory impairment in rat pups with hyperthermia-induced febrile seizures (Atabaki et al., 2020). Antiepileptic drug treatment is well considered to be associated with the modulation of cytokines. Valproic acid treatment in children with idiopathic generalized and partial epilepsy significantly reduced the serum levels of IL-10, when tested after 6 months of therapy (Jambaque et al., 1993). Hence, the anticonvulsant effect of hesperidin in the present study can also be correlated with a reduction in the IL-10 mRNA levels. Interestingly, in our study, PTZ treated group showed higher *il-10* expression than the group preincubated with hesperidin. These observations need to be investigated thoroughly in further studies.

The receptors for GABA and glutamate (NMDA receptors) are very crucial targets for antiepileptic drug research. Activation of NMDA receptors leads to calcium-calmodulin dependent CREB phosphorylation (Sharma et al., 2019). GABA neurotransmission is also associated with BDNF/TrkB activation. CREB–BDNF controls the development and operation of the GABAergic network, which again, in turn, impacts the BDNF level. Prolonged seizure activity alters the GABA_A_ receptor transcription and functions through a BDNF-dependent pathway (Grabenstrrater et al., 2012; Porcher et al., 2018). The present study showed the interaction of hesperidin with all these receptors. This interplay of NMDA and GABA receptors with CREB–BDNF/TrkB can be pertinent to the anticonvulsant mechanism of hesperidin. In the current study, it was inferred that citrus flavonoid, hesperidin, is actively involved in the modulation of CREB–BDNF pathway and shows anticonvulsant effect in the zebrafish larvae. However, the contribution of hesperidin and its aglycone form hesperitin in the overall pharmacological effect needs to be further investigated. Pharmacological characteristics of hesperidin action such as its effective and safe dosage, bioavailability profile, tolerability, potency and effect of its active metabolites are not known precisely. Although preclinical data is supporting the antiseizure and neuroprotective potential of this citrus flavonoid, however, well-planned clinical trials having rigorous designs are required to extrapolate the effects in human population.

## Conclusion

The current study’s findings concluded that hesperidin shows anticonvulsant action in the PTZ convulsion model of zebrafish. The results of the *in silico* computational analysis supported by gene expression studies showed the observed anticonvulsant effect through interaction with the central CREB–BDNF pathway. However, further studies are required to understand the interaction of hesperidin with cytokinins functions completely.

## Data Availability Statement

The raw data supporting the conclusions of this article will be made available by the authors on request, without undue reservation.

## Ethics Statement

The animal study was reviewed and approved by IAEC of CSIR-IHBT.

## Author Contributions

PS and SK performed zebrafish studies and helped in original draft writing; PS carried out RNA isolation and qRT-PCR-based gene expression studies; JK conducted *in silico* studies; RP conceptualized *in silico* studies and helped in original draft writing and; DS conceptualized the zebrafish study, performed data curation, original draft writing, reviewing and editing.

## Conflict of Interest

The authors declare that the research was conducted in the absence of any commercial or financial relationships that could be construed as a potential conflict of interest.
