# Supplementary material for: Hesperidin Interacts With CREB-BDNF Signaling Pathway to Suppress Pentylenetetrazole-Induced Convulsions in Zebrafish
Source: Front Pharmacol. 2021 Jan 11;11:607797. doi: 10.3389/fphar.2020.607797 (PMC7832091; doi:10.3389/fphar.2020.607797)
Supplement: Supplementary file 1 [file datasheet1.docx]

**Hesperidin interacts with CREB-BDNF signaling pathway to suppress pentylenetetrazole-induced convulsions in Zebrafish**


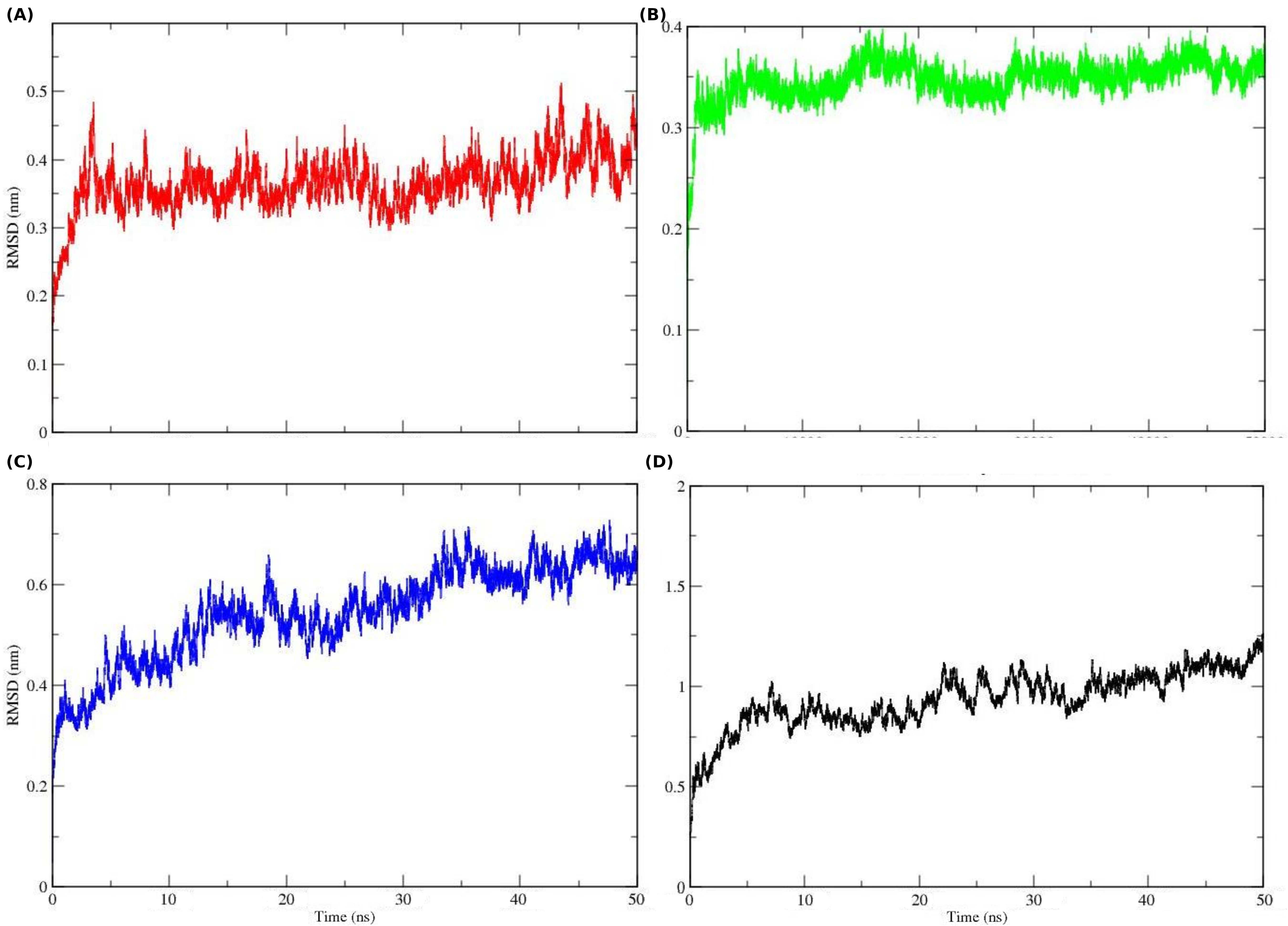


**Figure 1: Graphical representation of RMSDs. (A) NMDAR-Hesperidin; (B) TRK-Hesperidin; (C) GABA-Hesperidin and; (D) Interleukin-10-Hesperidin**


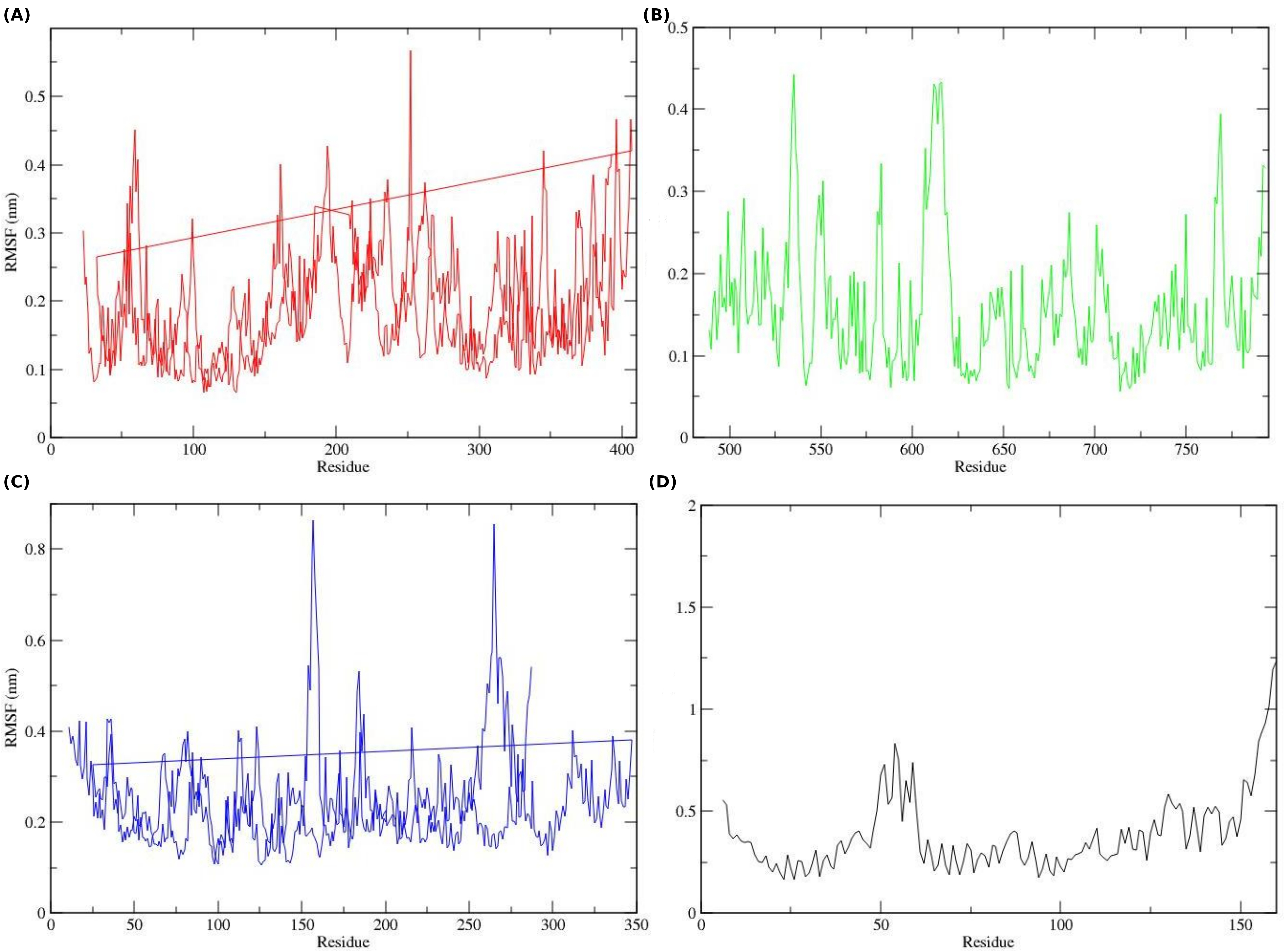


**Figure 2: Graphical representation of RMSF. (A) NMDAR-Hesperidin; (B) TRK-Hesperidin; (C) GABA-Hesperidin and; (D)Interleukin-10-Hesperidin**


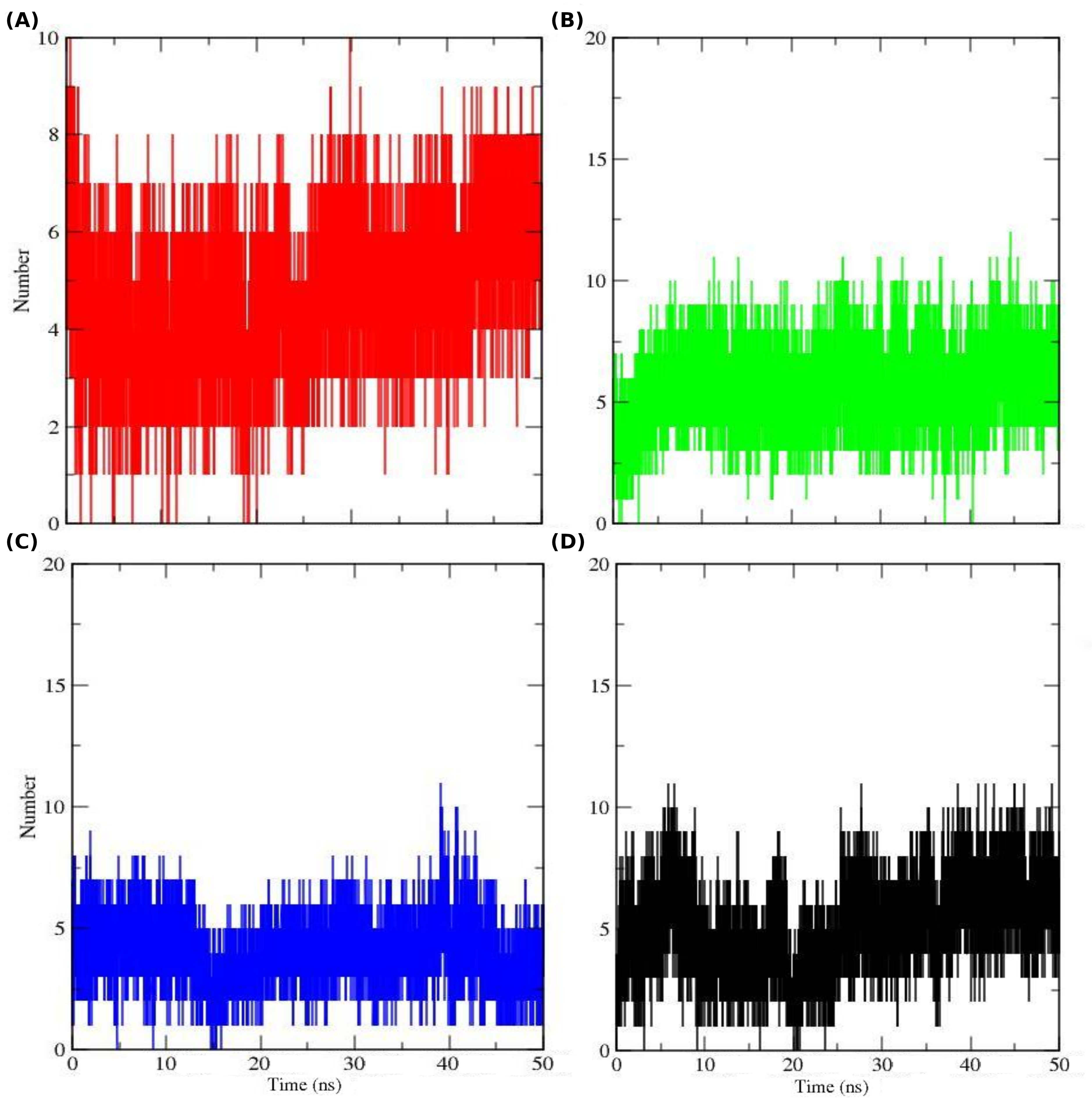


**Figure 3: Graphical representation of H-bonds between the receptors and hesperidin. (A) NMDAR-Hesperidin; (B)TRK-Hesperidin; (C)GABA-Hesperidin; and (D)Interleukin-10-Hesperidin**


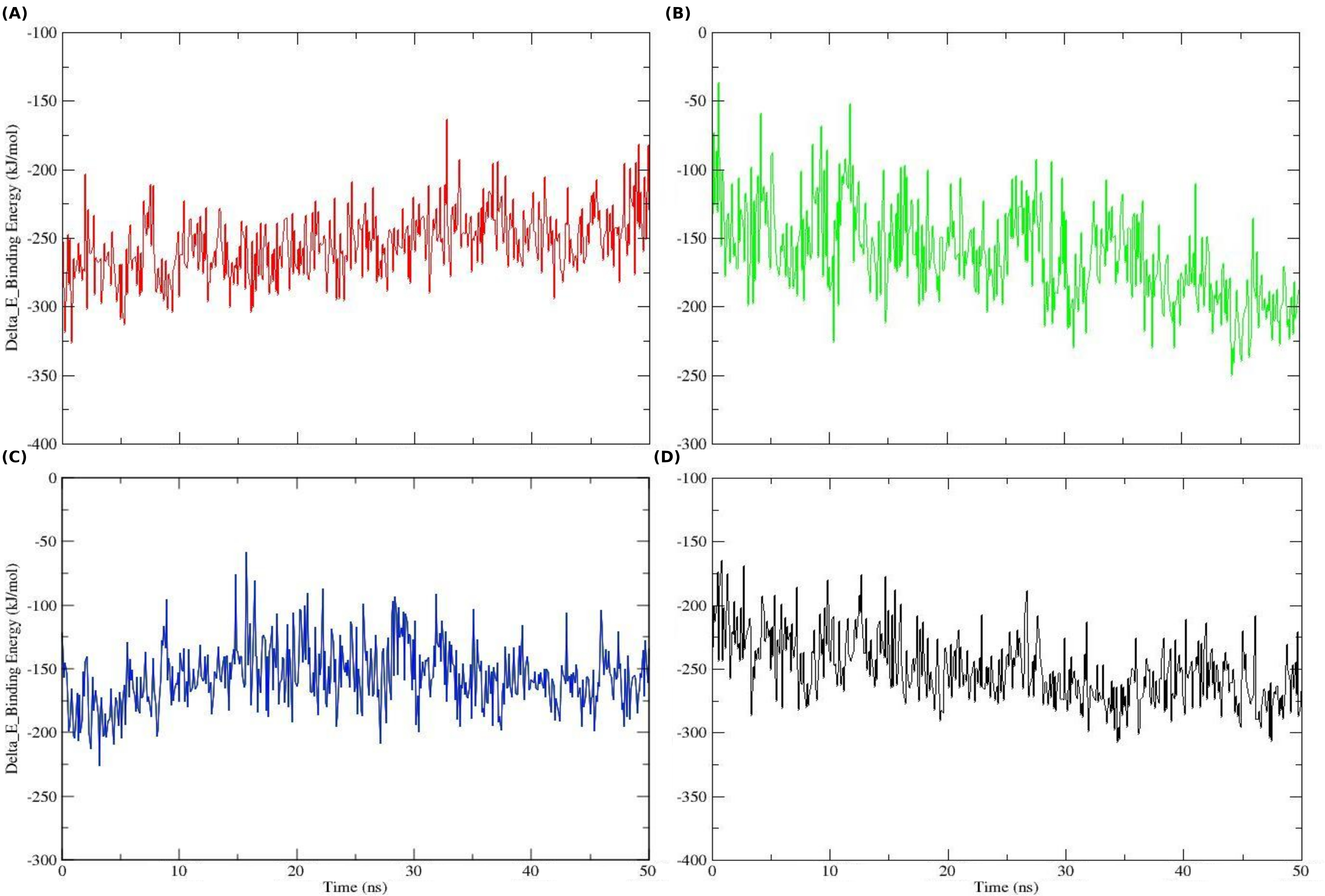


**Figure 4: Graphical representation of the binding energies obtained by hesperidin with the receptors. (A) NMDAR receptor; (B)TRK receptor; (C) GABA receptor and; (D) Interleukin-10 receptor**

**
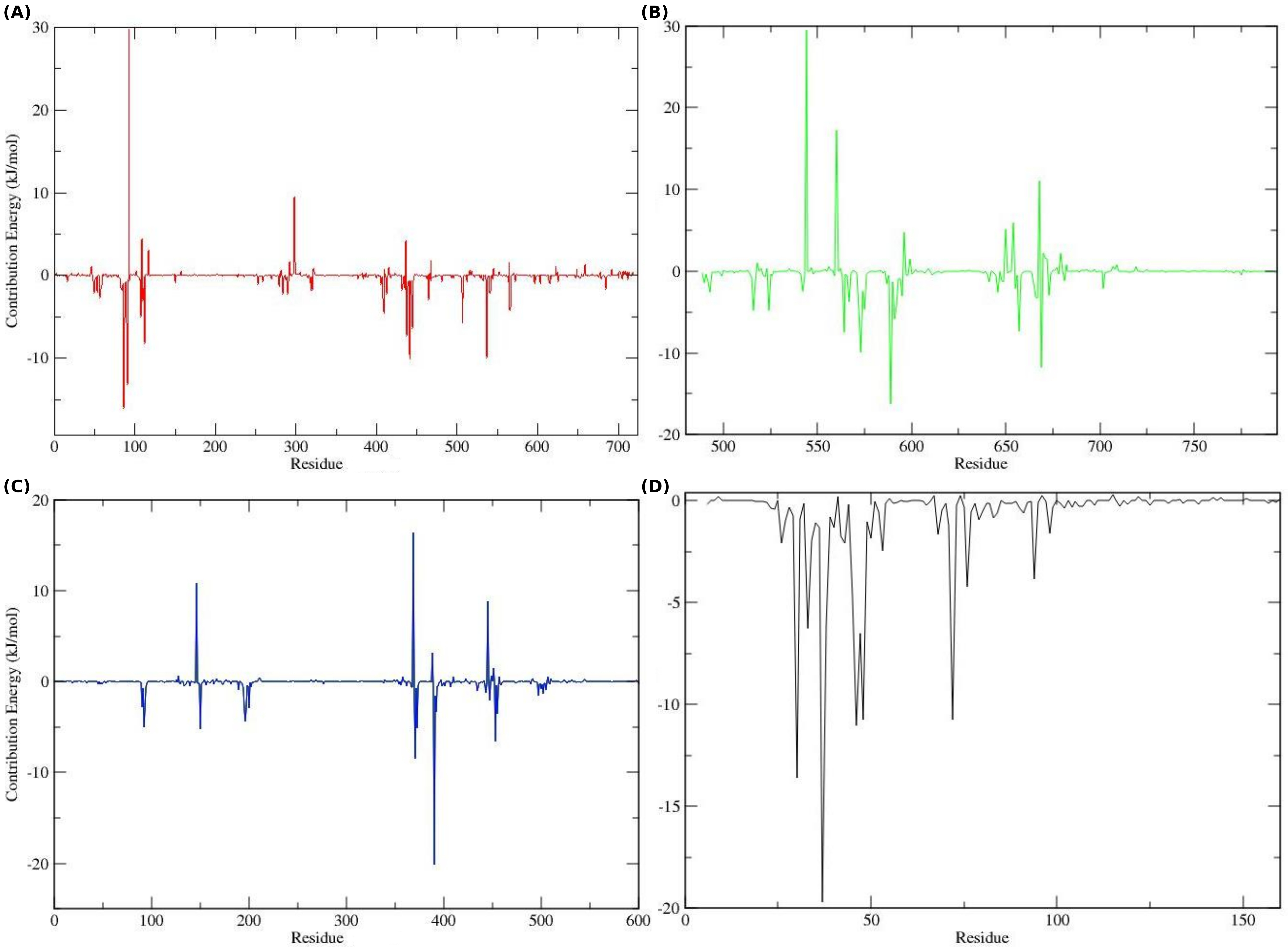
**

**Figure 5: Contribution energies of the residues of the protein receptor towards the hesperidin molecule. (A) NMDAR receptor; (B) TRK receptor; (C) GABA receptor and; (D) Interleukin-10 receptor**
